# The Antialgal Mechanism of Luteolin-7-O-Glucuronide on *Phaeocystis globosa* by Metabolomics Analysis

**DOI:** 10.3390/ijerph16173222

**Published:** 2019-09-03

**Authors:** Jingyi Zhu, Yeyin Yang, Shunshan Duan, Dong Sun

**Affiliations:** Department of Ecology, College of Life Science and Technology, Jinan University, Guangzhou 510632, China

**Keywords:** *Phaeocystis globosa*, luteolin-7-O-glucuronide, metabolomic analysis

## Abstract

Antialgal compounds from plants have been identified as promising candidates for controlling harmful algal blooms (HABs). In our previous study, luteolin-7-O-glucuronide was used as a promising algistatic agent to control *Phaeocystis globosa* (*P. globose*) blooms; however, its antialgal mechanism on *P. globosa* have not yet been elaborated in detail. In this study, a liquid chromatography linked to tandem mass spectrometry (LC-MS/MS)-based untargeted metabolomic approach was used to investigate changes in intracellular and extracellular metabolites of *P. globosa* after exposure to luteolin-7-O-glucuronide. Significant differences in intracellular metabolites profiles were observed between treated and untreated groups; nevertheless, metabolic statuses for extracellular metabolites were similar among these two groups. For intracellular metabolites, 20 identified metabolites showed significant difference. The contents of luteolin, gallic acid, betaine and three fatty acids were increased, while the contents of α-Ketoglutarate and acetyl-CoA involved in tricarboxylic acid cycle, glutamate, and 11 organic acids were decreased. Changes in those metabolites may be induced by the antialgal compound in response to stress. The results revealed that luteolin played a vital role in the antialgal mechanism of luteolin-7-O-glucuronide on *P. globosa*, because luteolin increased the most in the treatment groups and had strong antialgal activity on *P. globosa*. α-Ketoglutarate and acetyl-CoA were the most inhibited metabolites, indicating that the antialgal compound inhibited the growth through disturbed the tricarboxylic acid (TCA) cycle of algal cells. To summarize, our data provides insights into the antialgal mechanism of luteolin-7-O-glucuronide on *P. globosa*, which can be used to further control *P. globosa* blooms.

## 1. Introduction

The frequent occurrence of harmful algal blooms (HABs), which happens due to increasing water pollution and climate changes, is harmful to the environment, economy, and public health [1,2,3]. HABs can directly or indirectly poison aquatic animals and humans, discolor coastal waters and harm water quality, form fetid foam with unpleasant smell, damage light-dependent aquatic ecosystems, and hinder the tourism industry [4]. *Phaeocystis globosa* (*P. globosa*) is one of the most frequently reported HABs species and can produce monospecific blooms of gelatinous colonies [5,6]. It can also release toxic hemolytic substances and form nuisance foam, causing great damage to the marine ecosystem, tourism industry, human health, and agriculture [7,8]. Up until now, knowledge on how to prevent *P. globosa* blooms are still limited. Thus, in this study, *P. globosa* was selected as a representative specie of harmful algae.

The application of physical and chemical methods to prevent the outbreak of HABs has a wide range of shortcomings, including high cost, high toxicity, secondary pollution, and difficult implementation [9,10,11]. Additionally, some biological methods using living organisms, such as algae-fed aquatic birds or fish, algicidal bacteria, and viruses can be applied to control HABs; nevertheless, these methods lack technical stability and are likely to change or even destruct the current ecosystem [4,12]. Over the last decade, the application of antialgal compounds extracted from plants, such as *Ulva pertusa*, *Cinnamomum camphora*, *Arundo donax*, *Ascophyllum nodosum*, *Sagittaria trifolia*, *Stratiotes aloides*, and *Eichhornia crassipes* have been applied to control HABs; this approach has shown to be effective and has great potential to eliminate harmful algae, but to date, the antialgal mechanism is still not clear [13,14,15,16,17,18,19,20,21].

Luteolin-7-O-glucuronide is a flavone glycoside found in plants [22,23,24,25]. In our previous study, we discovered that luteolin-7-O-glucuronide extracted from *Enhalus acoroides* (*E. acoroides*) effectively inhibited the growth of harmful algae species *P. globosa* with EC_50–96h_ value of 34.29 μg/mL [26]. Luteolin-7-O-glucuronide possesses anti-microbial, antioxidant, anti-mutagenic, anti-genotoxic, anti-inflammatory, and anti-arthritic activities, which means that its application might be safe for human beings [27,28,29]. Moreover, the purified compound of luteolin-7-O-glucuronide is easily accessible for extraction and artificial synthesis since it can be found in a wide range of plants. These advantages imply that luteolin-7-O-glucuronide possesses good application potential as an algistatic agent. Nevertheless, it is still difficult to introduce the algistatic agent into practical application because the antialgal mechanisms are not fully elaborated.

Liquid chromatography linked to tandem mass spectrometry (LC-MS/MS)-based untargeted metabolomics has become a popular technique since they provide valuable information on overall changes in small metabolites and biochemical pathways in response to toxicants or environmental stressors [30]. In this study, we analyzed intracellular and extracellular metabolites of *P. globosa* after exposure to the antialgal compound by LC-MS/MS-based untargeted metabolomics analysis. The aim of this study was to further improve the application of antialgal compounds, such as luteolin-7-O-glucuronide, in order to control *P. globosa* blooms.

## 2. Materials and Methods

### 2.1. Algal Cultures

*P. globosa* were supplied by the Research Center of Hydrobiology, Jinan University, Guangzhou, China. The cultures were incubated in F/2 medium at around 25 °C under a photoperiod of 12 h (light):12 h (dark) with a light intensity of 100 μmol photons m^−2^s^−1^. *P. globosa* culture in the exponential growth phase was used for the experiments.

### 2.2. Antialgal Assays

Luteolin-7-O-glucuronide (96% purity) were purchased from Chengdu Push Bio-Technology Co., Ltd. (Chengdu, China). The solution of the compound was dissolved in F/2 medium prepared with seawater and filtered with 0.22 μm filter before use. *P. globosa* (1 × 10^5^ cells/mL) was treated with 34.29 µg/mL luteolin-7-O-glucuronide for 48 h. The algal cultures that were not treated served as controls. It can be seen from Figure S1, the growth of *P. globosa* was significantly inhibited after 48 h exposure, indicating the antialgal compound had exerted its inhibitory effects at that time. Some metabolites can be degraded under long-term illumination, which may have adverse effects on the monitoring of metabolites. Additionally, cell viability analysis was performed, and the method was described in the supplementary files. According to the cell integrity of *P. globosa* (Figure S2), only 12.8% *P. globosa* was killed by the antialgal compound at 48h, but the percentage of dead cells for 96h was 54.33%. The contents of extracellular metabolites may influence by intracellular metabolites that were released from dead cells into the medium. Therefore, algal cells treated with 34.29 µg/mL luteolin-7-O-glucuronide for 48 h were used to perform metabolomic analysis.

### 2.3. LC-MS/MS-Based Untargeted Metabolomic Analysis

#### 2.3.1. Metabolites Extraction

In this step, 50 mL of culture was taken out each from the six independent control and treated groups. Then, the cells were collected by 5 min centrifugation under 14,000× *g* at 4 °C. The cell samples were used for intracellular metabolomic analysis and the supernatant samples were used for extracellular metabolomic analysis. The cell samples (100 mg) in both control and treated groups were individually grounded with liquid nitrogen and 100 μL of the homogenates was resuspend with precooled 80% methanol at −20 °C followed by vortexing. The supernatant samples (100 μL) were also grounded with liquid nitrogen (−20 °C) and 400 μL 80% methanol was added followed by vortexing. All samples were incubated at −20 °C for 60 min and then were centrifuged at 14,000× *g*, 4 °C for 15 min. Then the supernatants were transferred to a fresh microcentrifuge tube and dried under vacuum in a centrifugal evaporator. Before metabolomic analysis, the dried metabolite pellets were dissolved by 80% methanol. The equivalent supernatant was mixed from each processed sample as QC (Quality control) samples.

#### 2.3.2. LC-MS/MS Condition

LC-MS/MS analyses were carried out on a Vanquish UHPLC system (Thermo Fisher, Waltham, MA, USA) coupled with an Orbitrap Q Exactive HF-X mass spectrometer (Thermo Fisher, Waltham, MA, USA) operating in the data-dependent acquisition (DDA) mode. Samples were injected into an Accucore HILIC column (100 × 2.1 mm, 2.6 μm) with a 20-min linear gradient at a flow rate of 0.3 mL/min. The following solvent gradient was used: 2.0% B, 1.0 min; 2.0–50.0% B, 16.5 min; 50.0–2.0% B, 2.5 min. Q-Exactive HF-X mass spectrometer was operated with spray voltage of 3.2 kV, sheath gas flow rate of 35 arb, capillary temperature of 320 °C, and aux gas flow rate of 10 arb. The raw data files were processed using the Compound Discoverer 3.0 (CD 3.0, Thermo Fisher, Waltham, MA, USA) to perform peak alignment, peak picking, and quantitation for each metabolite. The main parameters were as follows: retention time tolerance, 0.2 min; actual mass tolerance, 5 ppm; signal intensity tolerance, 30%; signal/noise ratio, 3; and minimum intensity, 100,000. Peak intensities were normalized to the total spectral intensity and the data were normalized to predict the molecular formula based on additive ions, molecular ion peaks, and fragment ions. Peaks were matched with the mzCloud and ChemSpider database to obtain the accurate qualitative and relative quantitative results and all samples were uploaded and searched in the database together.

#### 2.3.3. Data Analysis

The metabolite experiment was conducted in six independent samples. The data were filtered by CV (coefficient of variance), retaining the metabolites of CV <0.3. We applied univariate analysis to calculate the statistical significance (*p*-value) and fold change (FC) of the metabolites between two group means. After log2 treatment, the relative quantitative value of the differential metabolites satisfied the normal distribution and then a two-tailed t-test was used. To maximize identification of differences in metabolites between two groups, a supervised method of PLS-DA (partial least squares discriminant analysis) was used to maximize the difference of metabolites; metabolites were selected using PLS-DA with variable importance in the projection score (VIP). Metabolites were identified to be responsible for the separation for their VIP with a threshold greater than 1. Statistical analyses were performed using the statistical software R (R version R-3.4.3), Python (Python 2.7.6 version). Principal Component Analysis (PCA) and clustering analysis were performed by R software. A volcano plot of negative log10-transformed *p*-values against the log2 fold change was used to illustrate a large number of metabolites between the treatment groups and the control groups by GraphPad Prism 7 software [31].

## 3. Results

### 3.1. PCA Analysis and Clustering Analysis of LC-MS/MS Metabolomics Profiles of P. Globosa

**Figure 1:**
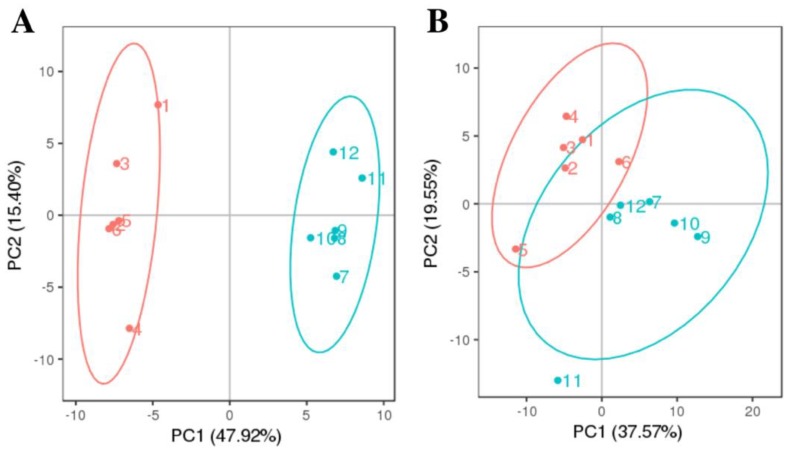
Principal Component Analysis (PCA) score plots of (**A**) intracellular metabolites and (**B**) extracellular metabolites. Red circles (1–6) represent untreated *P. globosa* cells; blue circles (7–12) represent *P. globosa* cells treated with 34.29 μg/mL luteolin-7-O-glucuronide for 48 h. The confidence ellipse is 95%.

LC-MS/MS-based untargeted metabolomic approach was employed to investigate changes in intracellular and extracellular metabolites between treated and untreated groups. The correlation of QC samples (closer to 1) indicates that the method was reliable and reproducible, and the data were of high quality for further data analysis (Figure S3). Briefly, 150 metabolites in medium (Table S1) and 106 metabolites in *P. globosa* cells (Table S2) were detected in both treated and untreated groups and identified. PCA analysis was performed in order to reduce the dimensionality of the data and to visualize samples grouping (Figure 1). The discrimination on the PCA profile for intracellular metabolites reflected the metabolic difference caused by the antialgal compound; the first principle component (PC1) explained 47.92% of the total variance, while the second principal component (PC2) 15.40% of the total variance. As for PCA score plot of extracellular metabolites, PC1 and PC2 covered 37.57% and 19.55%, respectively. Two groups for extracellular metabolites were located together, which indicated that metabolic statuses of those groups were similar. To investigate the changes after exposure to luteolin-7-O-glucuronide, the PCA loading plot of intracellular metabolites and extracellular metabolites corresponding to the PCA score plot were generated (Figure S4). Luteolin was identified as main drivers of for the sample separation in both intracellular and extracellular metabolites.

**Figure 2:**
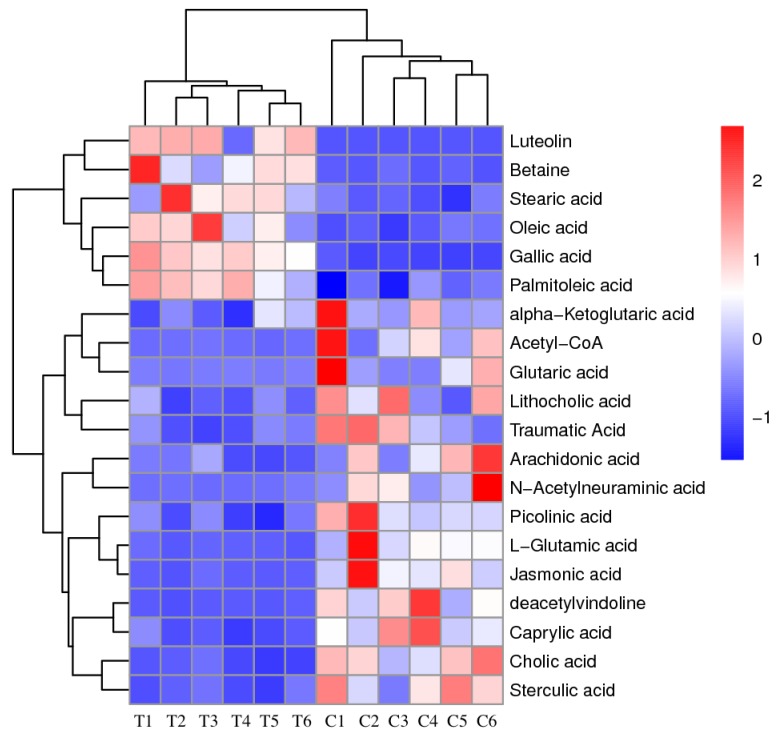
Heat map generated by hierarchical cluster analysis.

The relative abundances of fluctuated intracellular metabolites in all groups are presented in the heat map and two major clusters were identified (Figure 2). Metabolites that showed a decreased level included acetyl-CoA, alpha-ketoglutaric acid, L-glutamic acid and some organic acids, forming the first cluster. Metabolites that increased in algal cells compared with the control groups formed the second cluster including luteolin, gallic acid, betaine, and three fatty acids. The patterns of metabolite clustering clearly illustrate the metabolic changes under exposure to the antialgal compound.

### 3.2. Volcano Plot Analysis of LC-MS/MS Metabolic Profiles of P. globosa

**Figure 3:**
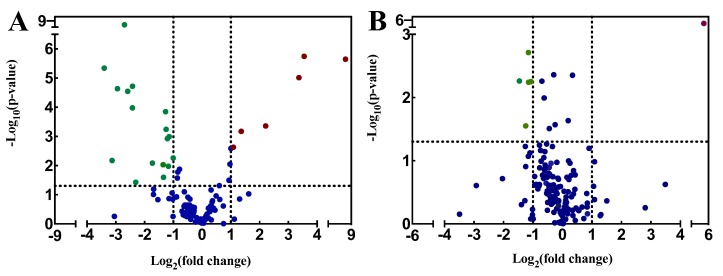
Volcano plot of metabolites of *P. globosa* after exposure to 34.29 μg/mL luteolin-7-O-glucuronide for 48 h. (**A**) Intracellular metabolites; (**B**) extracellular metabolites. Red points represent upregulated metabolites. Green points represent downregulated metabolites. Blue points indicate no significant differences.

The volcano plots were used to show qualitative information of the metabolic datasets (Figure 3). For intracellular metabolites, 6 metabolites increased and 14 decreased compared to the control group. Meanwhile, the concentrations of luteolin, gallic acid, and betaine were most increased in the treatment groups, and were 365.15-fold, 11.72-fold and 10.33-fold as compared to the control groups, respectively. The α-Ketoglutarate, acetyl-CoA, and L-glutamic acid were the most inhibited metabolites; their concentrations were only 9.45%, 11.42%, and 12.01% of that in control, respectively. On the basis of the parameter VIP score, 12 intracellular metabolites were considered to be responsible for explaining the responses for their VIP score greater than 1 (Table 1). Nevertheless, less regulated extracellular metabolites were found in medium; among the extracellular metabolites, 1 increased and 4 decreased compared to the control group.

## 4. Discussion

With the outbreak of *P. globosa* blooms, the application of antialgal compounds to control HABs has become a topic of interest within the scientific community. In our previous study, luteolin-7-O-glucuronide showed great potential as antialgal compound to control HABs [26]. Nevertheless, the antialgal mechanism has not yet been fully elaborated. In this study, the antialgal mechanism of luteolin-7-O-glucuronide on *P. globosa* was explored using metabolomics analysis, which provided a deeper insight into the response of algae to antialgal compounds.

LC-MS-based metabolomics have been used to examine extracellular and intracellular metabolomic profiles of *P. globosa* under treatment of antialgal compound luteolin-7-O-glucuronide. The results showed similar extracellular metabolites profiles between treated and untreated group, while clear difference was observed in relation to intracellular metabolomic profiles. We speculate that most of the intracellular metabolites that are over-produced inside the live cell would not be secreted and intracellular metabolites secreted from 12.8% dead cells had a small effect on the contents of extracellular metabolites (Figure S1), eventually resulting in the less regulated extracellular metabolites [32].

The metabolite luteolin was up regulated in both cells and medium. The up regulation of luteolin may be partially related to instability of the glucuronide, but the main reason for upregulated luteolin was β-glucosidases that are found in almost all organisms to hydrolyze glycosidic bonds of luteolin-7-O-glucuronide [33]. It has been reported that luteolin significantly inhibited the growth of *P. globosa* and the inhibition rate was over 80% after three days of exposure [34]. Thus, we speculated that luteolin played a vital role in inhibiting the growth of *P. globosa* in our study. In the present study, we found that some fatty acids, such as stearic acid, palmitoleic acid, and oleic acid were all upregulated in the treatment group, indicating the elevation of fatty acid biosynthesis metabolism. Previous studies have shown that plants can change the fatty acid composition when exposed to environmental stress [35,36]. These findings were consistent with what we found in *P. globosa* after exposure to the antialgal compound. Additionally, another metabolite, betaine was found to be up regulated in algal cells, which is an organic osmolyte that contributes to osmoregulation and osmoprotection [37].

Furthermore, we found that alpha-Ketoglutaric acid was downregulated, and acetyl-CoA were downregulated in the treatment group compared to the control samples. Both metabolites are involved in the tricarboxylic acid cycle (TCA cycle). The TCA cycle not only provides energy for life activities but also produces a wide range of intermediate products that are raw materials for biosynthesis of many important substances in the organisms [38]. Therefore, we inferred that the antialgal compound inhibited the growth of algal cells through disturbed the TCA cycle of algal cells, which was consistent with our previous study that mitochondrion where the TCA cycle occurred was seriously damaged by the antialgal compound under transmission electron microscopy [26]. Mitochondria is used to generate energy required for cellular processes by oxidizing organic acids in the TCA cycle [39]. We also observed the downregulation of L-glutamic acid (Glu) in algal cells. Glu takes part in the biosynthesis of alanine, valine, and leucine involved in the transamination of the amino moiety [40]. It has been reported that the concentration of Glu in *Chlorella vulgaris* decreases after exposure to heavy metal (copper) in high concentrations, which is consistent with our study [37]. There were many types of organic acids that have also shown to be downregulated in algal cells. A decreased level of organic acids was also found in wheat when exposed to stress [30].

## 5. Conclusions

In this study, the effects of luteolin-7-O-glucuronide on the metabolomic changes of *P. globosa* were investigated. We found different intracellular metabolites profiles, but similar extracellular metabolites after exposure to luteolin-7-O-glucuronide. Overall, 20 intracellular metabolites showed a significant difference. Changes in those metabolites may be induced by the antialgal compound in response to stress. This finding provides valuable information on the antialgal mechanisms of luteolin-7-O-glucuronide on *P. globosa*.

The application of luteolin-7-O-glucuronide is effective, might be safe for human beings, and is easily accessible for extraction and artificial synthesis. However, further studies are needed to fully elucidate the toxicity of the antialgal compound for non-target organisms to maintain the stability of ecosystem. Additionally, it is of great practical significance to develop a reasonably priced method of synthesizing luteolin-7-O-glucuronide in controlling *P. globosa* blooms.

## Figures and Tables

**Table 1:** Upregulated and downregulated intracellular metabolites after exposure to luteolin-7-O-glucuronide in *P. globosa.* VIP: variable importance in the projection score.

Metabolites	Log_2_ (Fold Change)	−Log_10_ (*p* Value)	VIP	Up/Down
Acetyl-CoA	−3.13	2.17	1.95	down
L-Glutamic acid	−2.94	4.64	1.62	down
alpha-Ketoglutaric acid	−3.40	5.34	1.91	down
Arachidonic acid	−1.36	2.03	0.73	down
Deacetylvindoline	−2.42	3.98	1.33	down
Glutaric acid	−2.31	1.43	1.25	down
Jasmonic acid	−2.59	4.55	1.43	down
Lithocholic acid	−1.34	1.60	0.72	down
Caprylic acid	−1.27	3.85	0.71	down
Cholic acid	−2.42	4.72	1.81	down
N-Acetylneuraminic acid	−1.73	2.08	1.10	down
Picolinic acid	−1.14	2.99	0.66	down
Sterculic acid	−1.21	2.92	0.85	down
Traumatic acid	−1.17	1.97	0.63	down
Luteolin	8.51	5.65	4.78	up
Gallic acid	3.55	5.74	2.08	up
Oleic acid	2.21	3.36	1.64	up
Betaine	3.37	5.01	1.91	up
Palmitoleic acid	1.09	2.63	0.66	up
Stearic acid	1.36	3.17	0.97	up

## Supplementary Materials

The following are available online at https://www.mdpi.com/1660-4601/16/17/3222/s1, Figure S1: The inhibition rate of P. globosa treated with 34.29 μg/mL luteolin-7-O-glucuronide, Figure S2: Cell integrity of P. globosa with and without exposure to 34.29 μg/mL luteolin-7-O-glucuronide, Figure S3: Pearson correlation between QC samples, Figure S4: PCA loading plots of (A) intracellular metabolites and (B) extracellular metabolites, Table S1: The list of identified metabolites in medium, Table S2: The list of identified metabolites in algal cells.
