# Supplementary material for: The Antialgal Mechanism of Luteolin-7-O-Glucuronide on Phaeocystis globosa by Metabolomics Analysis
Source: Int J Environ Res Public Health. 2019 Sep 3;16(17):3222. doi: 10.3390/ijerph16173222 (PMC6747131; doi:10.3390/ijerph16173222)
Supplement: Supplementary file 1 [file ijerph-16-03222-s001.zip › supplementary files for publication.docx]

Supplementary Files: The Antialgal Mechanism of Luteolin-7-O-Glucuronide on *Phaeocystis Globosa* by Metabolomics Analysis

Jingyi Zhu, Yeyin Yang, Shunshan Duan *, and Dong Sun *

Department of Ecology, College of Life Science and Technology, Jinan University, Guangzhou 510632, China

***** Correspondence: tssduan@jnu.edu.cn (S.D.); jnu_sundong@163.com (D.S.); Tel.: +86-(020)-8522-3192 (D.S.); +86-135-3554-8071 (S.D.)

1. Method of Cell Viability Analysis

A flow cytometer (BD FACSAria, Heidelberg, Germany) was employed for determining cell integrity of *P. globosa*. Briefly, a red fluorescence detector (FL4 detector, 650 nm) was used to detect the auto-fluorescence from chlorophyll in the algal cells and a standard fluorescence detector (FL2 detector, 610 nm) was used to detect algal cells stained with Propidium iodide (PI). The samples were stained with PI (a nucleic acid stain) at a final concentration of 10 µmol/mL and then incubated for 15 min at room temperature. The flow rates of algal cells were set at 100–400 cells s^−1^. Data were recorded on a logarithmic scale for each analyzed parameter.

2. Data Analysis for Figures S1–S2

Statistical analysis of data was conducted using GraphPad Prism 7 software (GraphPad Software, Inc., San Diego, CA). The treated groups compared the difference with the controls for each time point. The means and standard deviations (SD) of all data were determined and graphed. Students’ t-test was used, and *p* < 0.05 and *p* < 0.01 were considered significant.

**Figure S1.** The inhibition rate of *P. globosa* treated with 34.29 μg/mL luteolin-7-O-glucuronide. Data are means ± SD (n = 3). * *p* < 0.05, ** *p* < 0.01 indicate significant differences.

**Figure S2.** Cell integrity of *P. globosa* with and without exposure to 34.29 μg/mL luteolin-7-O-glucuronide. Data are means ± SD (n = 3). * *p* < 0.05, ** *p* < 0.01 indicate significant differences.


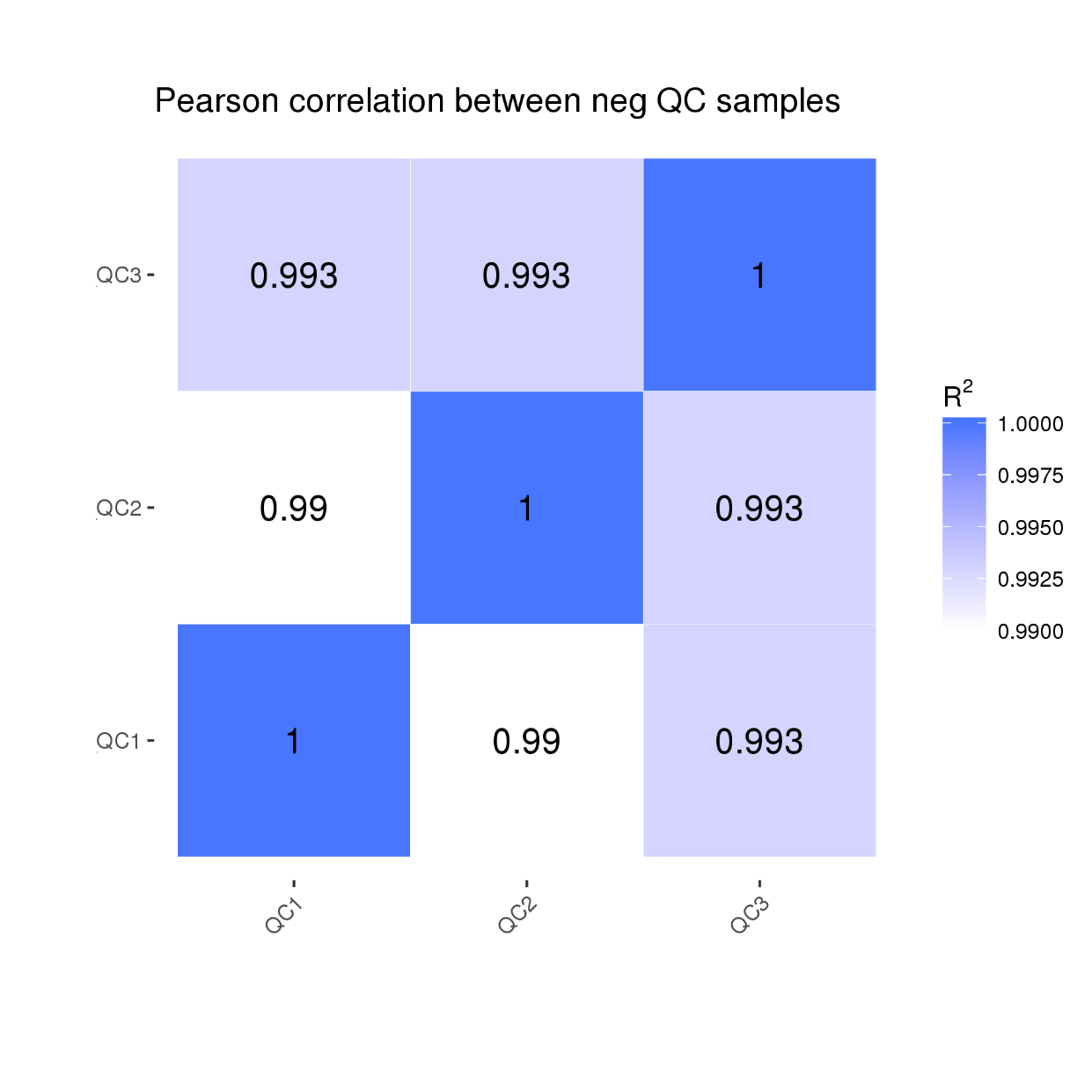


**Figure S3.** Pearson correlation between QC (Quality control) samples.

**
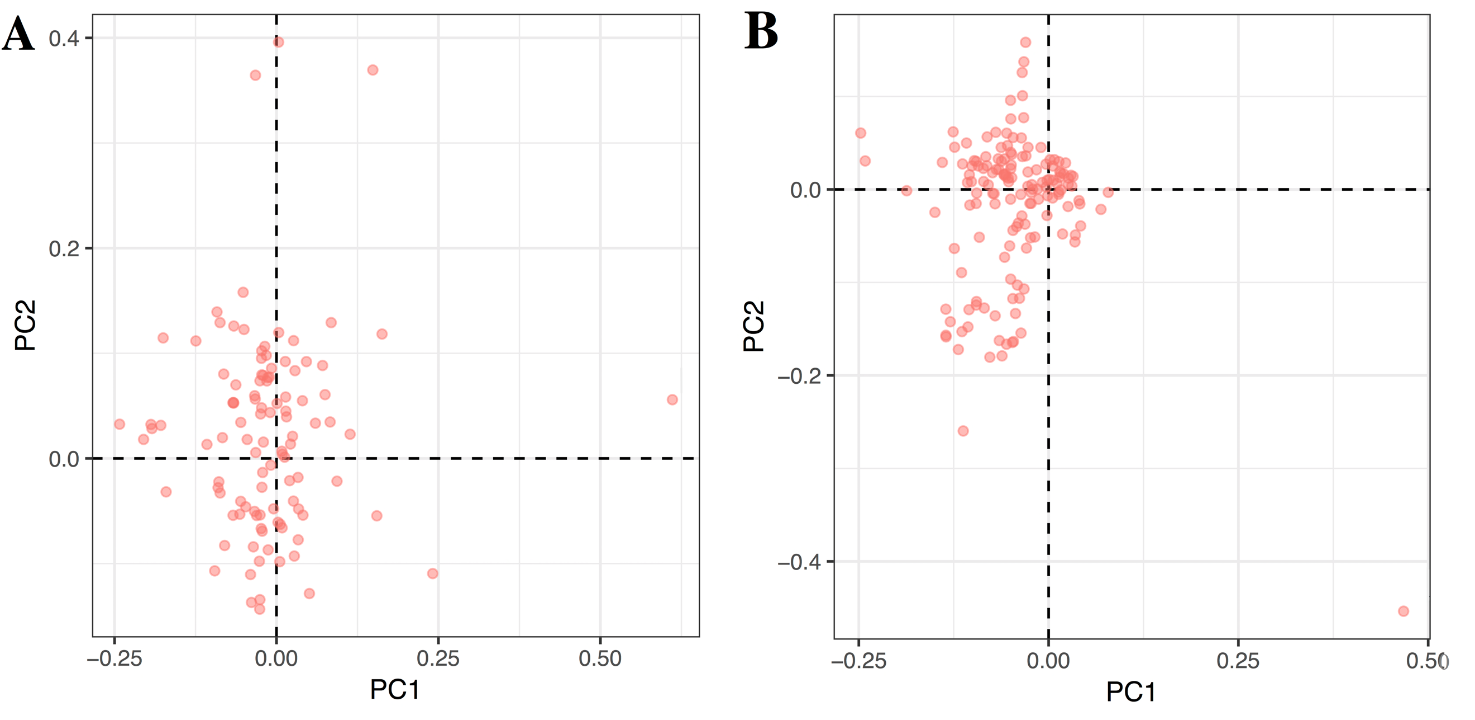
Figure S4.** PCA (Principal Component Analysis) loading plots of (**A**) intracellular metabolites and (**B**) extracellular metabolites.
