# Supplementary material for: The Antialgal Mechanism of Luteolin-7-O-Glucuronide on Phaeocystis globosa by Metabolomics Analysis
Source: Int J Environ Res Public Health. 2019 Sep 3;16(17):3222. doi: 10.3390/ijerph16173222 (PMC6747131; doi:10.3390/ijerph16173222)
Supplement: Supplementary file 1 [file ijerph-16-03222-s001.zip › ╨┬╜¿╬─╝■╝╨/S materials.docx]

*1.Method of ﻿cell viability analysis：*

A flow cytometer (BD FACSAria) was employed for determining cell integrity of *P. globosa*. Briefly, a red fluorescence detector (FL4 detector, 650 nm) was used to detect the auto-fluorescence from chlorophyll in the algal cells and a standard fluorescence detector (FL2 detector, 610 nm) was used to detect algal cells stained with Propidium iodide (PI). The samples were stained with PI (a nucleic acid stain) at a final concentration of 10 µmol/mL and then incubated for 15 min at room temperature. The flow rates of algal cells were set at 100–400 cells s^−1^. Data were recorded on a logarithmic scale for each analyzed parameter.

*2. Data analysis for Figures S1-S2*

Statistical analysis of data was conducted using GraphPad Prism 7 software. The treated groups compared the difference with the controls for each time point. The means and standard deviations (SD) of all data were determined and graphed. Students’ t-test was used, and *p* < 0.05 and *p* < 0.01 were considered significant.

**Figure S1.** The inhibition rate of *P. globosa* treated with 34.29 μg/mL luteolin-7-O-glucuronide. Data are means ± SD (n = 3). * *p* < 0.05, ** *p* < 0.01 indicate significant differences.

**Figure S2.** Cell integrity of *P. globosa* with and without exposure to 34.29 μg/mL luteolin-7-O-glucuronide. Data are means ± SD (n = 3). * *p* < 0.05, ** *p* < 0.01 indicate significant differences.


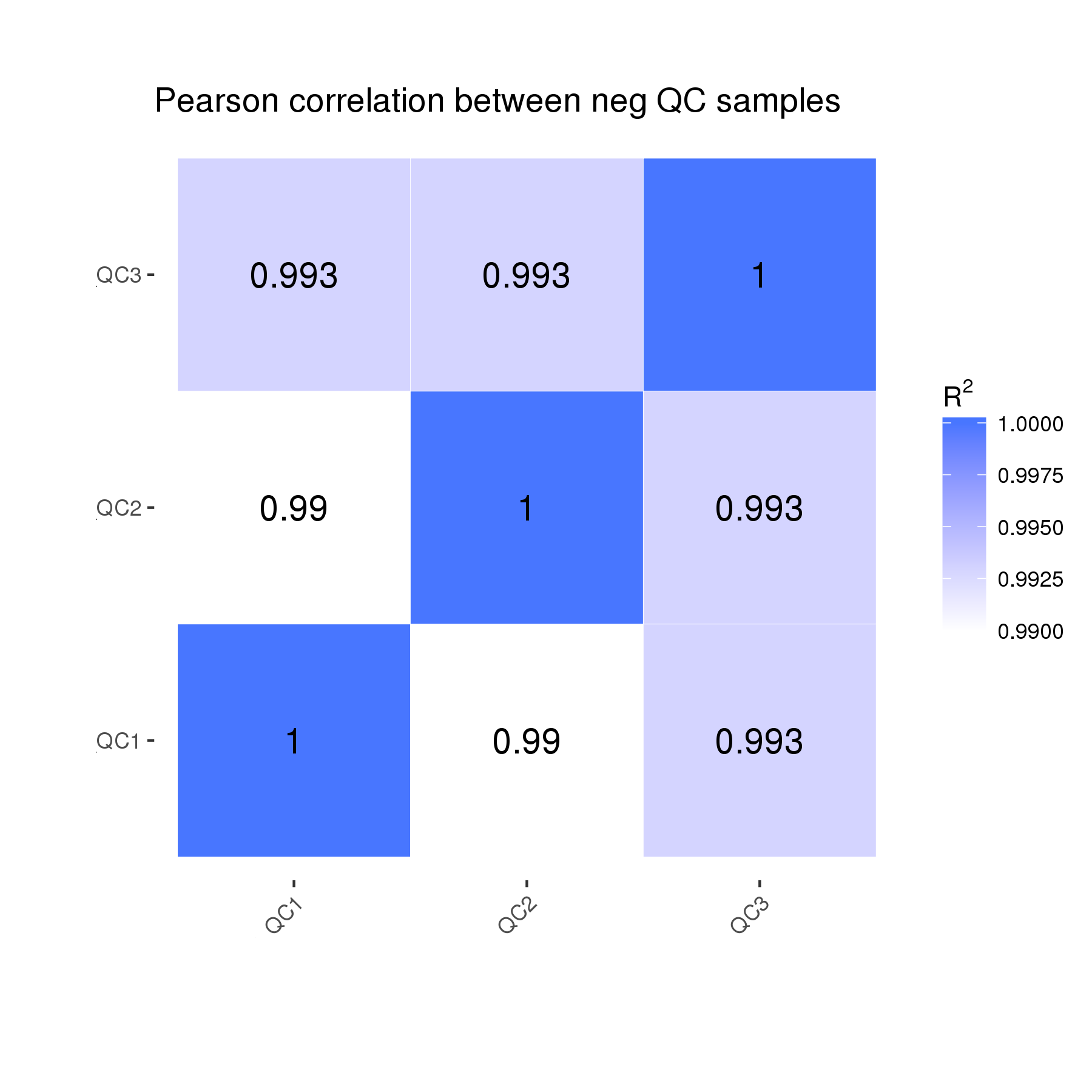


**Figure S3.** Pearson correlation between QC samples.


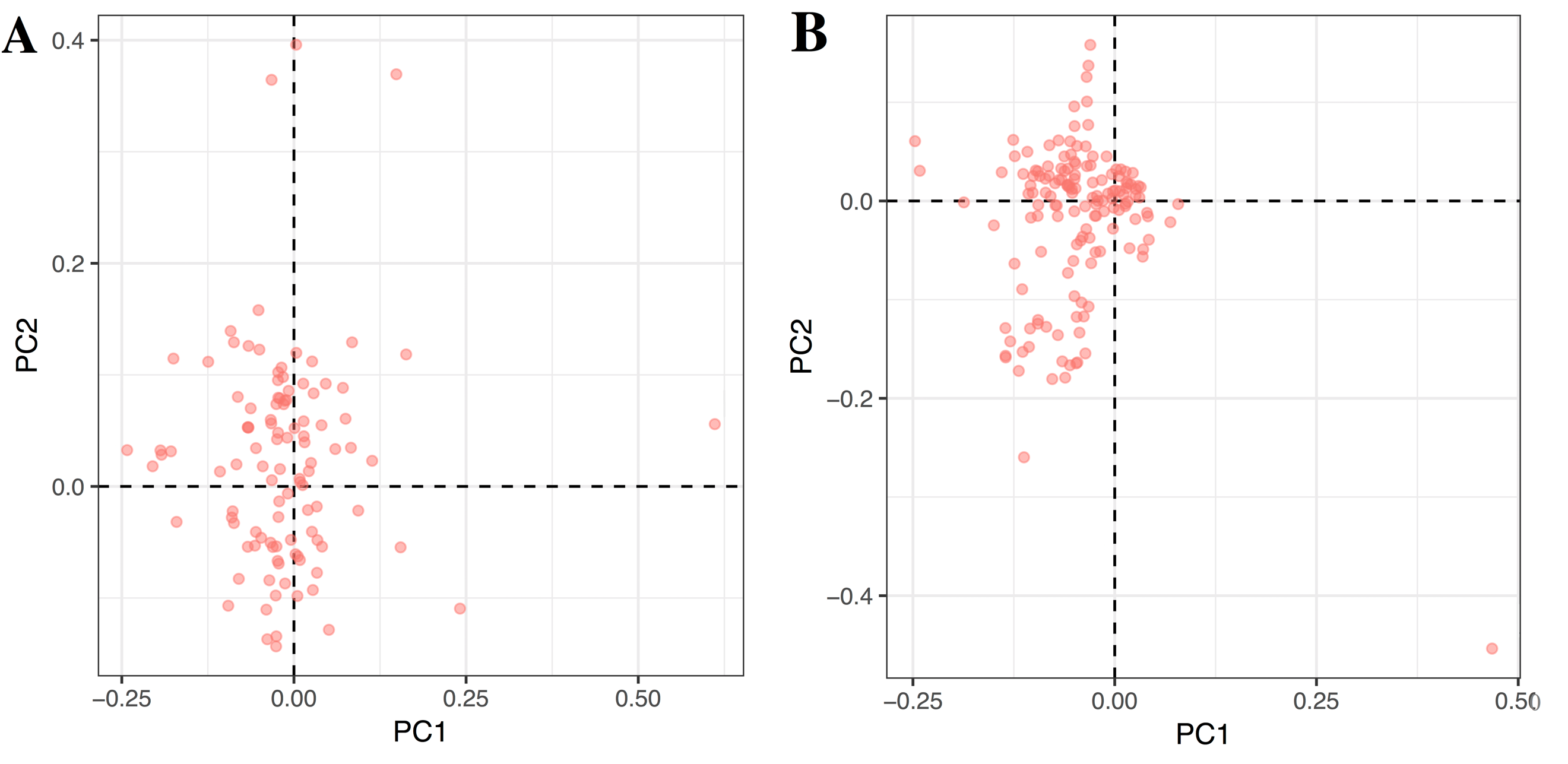
**Figure S4.** PCA loading plots of (A) intracellular metabolites and (B) extracellular metabolites.
